# A meta-narrative review of coding tools for healthcare interactions and their applicability to written communication

**DOI:** 10.1016/j.pecinn.2023.100211

**Published:** 2023-09-08

**Authors:** Elena Rey Velasco, Hanne Sæderup Pedersen, Ditte Hjorth Laursen, Timothy Skinner

**Affiliations:** a Department of Psychology, Faculty of Social Sciences, University of Copenhagen, Øster Farimagsgade 2A, 1353 Copenhagen K, Denmark; b Liva Healthcare, Danneskiold-Samsøes Allé 41, 1434, Copenhagen K, Denmark

**Keywords:** Communication, Patient-provider interaction, Telehealth, Empathy, Linguistics

## Abstract

**Background:**

Although healthcare professionals (HCP) undergo communicative skills training, these are sometimes unsatisfactory for patients (empathy, discussion managing). Existing coding tools overlook the interaction and patients' responses. Meanwhile, remote consultations are redefining communication channels. While some researchers adapt those tools to telehealth, few investigate written interactions.

**Objective:**

To identify and evaluate coding tools for healthcare interactions and examine their suitability for written interactions.

**Methods:**

We conducted a meta-narrative review in PubMed, PsycINFO, Embase, Web of Science, CINAHL, and Scopus databases up to December 2022 with Communicati* AND Human* AND Linguistic* AND Professional-Patient Relation* as search terms. We extracted data regarding methodology, unit of analysis (UoA), coding categories, reliability, strengths, weaknesses, and inter-rater reliability (IRR).

**Results:**

We identified 11 mixed-methods tools. Qualitatively, coding dimension was focused (*n* = 6) or comprehensive (*n* = 5). Main quantitative methods were descriptive statistics (*n* = 4) and cross-tabulations (n = 4). Main UoA was utterance (*n* = 7). Relevant categories were processes (n = 4), content (*n* = 3), emotional expressions and responses (n = 3), and grammatical format (*n* = 2). IRR ranged from 0.68 to 0.85 for coding categories.

**Conclusion:**

Despite similarities, category terminologies were inconsistent, one-sided, and mostly covered conversation topics and behaviours. A tool with emotional and grammar categories could bridge the gap between a speaker's intended meaning and the receiver's interpretation to enhance patient-HCP communication. Furthermore, we need empirical research to determine whether these tools are suitable for written interactions.

**Innovation:**

This review presents a comprehensive and state-of-the-art overview of healthcare interactions' coding tools and identifies their barriers. Our findings will support communication researchers in selecting appropriate coding tools for evaluating health interactions and enhancing HCP training.

## Highlights

• Coding tools lack thorough coverage of patients' emotional expressions and responses.
• Implications of telehealth and its compatibility with existing tools need exploration.
• We reviewed 11 coding tools and examined their suitability for written interactions.
• Coding categories (conversation topics, behaviours) across tools were heterogeneous.
• Our empathy + language approach augments tools and applies to written interactions.

## 1 Introduction

Patient-provider communication is key in any healthcare interaction. While a positive relationship between a patient and a healthcare professional (HCP) results in better outcomes [1], a good clinical interaction improves information recall after the consultation, the individual's trust in the HCP and, consequently their adherence to the recommendations and counselling received [2]. These effects are extremely important in the management of chronic conditions, such as diabetes or cardiovascular diseases, which can be improved or even reversed through health-related behaviour change interventions [3].

The relationship built with a patient relies heavily on the HCP's social and communicative skills [4]. During their education, HCPs undergo communicative skills training, including topics such as communication function, building relationships, gathering information, providing information, making decisions, responding to emotions, managing uncertainty, and enabling patient self-managing [5]. Extensive research shows that these skills are frequently unsatisfactory when assessed in clinical practice [6] and from the patients' perspective [7,8]. Some examples involve missed opportunities by the HCP to show empathy or to start a relevant discussion with the patient [9]. While there are several coding systems to assess HCP's communication skills [10,11] or to evaluate their relationship building techniques [12,13], these do not examine how the communicative competencies are delivered, such as turn-taking behaviour, or consider how their actions are received and interpreted. A complete understanding of the patient-provider interaction is fundamental to identify the most challenging elements in the communication, such as non-verbal behaviour and poor listening [14], and how to overcome these.

Several authors have developed and described qualitative coding systems that focus on the topics of patients' verbal and non-verbal behaviours [15] and speech topics [16], as well as consultation length [17]. Traditionally, verbal interaction coding entails three phases: recording and transcription/transfer of the conversation to a coding software, segmentation of this conversation into codable units, and allocation of coding categories to these units [18]. The coding categories typically undergo a quantitative analysis to define frequencies and draw conclusions, for example, from their link with patient outcomes and characteristics [19]. These coding systems have produced relevant insights for healthcare interactions, such as consultation topics [20] and common misunderstandings [21], that represent the basis of a growing body of research. However, the missing element in these coding systems is often the dynamic nature of the interaction and the study of what happens during the actual interaction and the linguistic exchange [22]. A conversation is an interactive and cooperative process. The speakers shape the interaction as they communicate according to their needs, and exchange different meanings through their social and cognitive resources, for example, during an interview with questions and answers [23]. An utterance is a single unit of communication (word or statement) that is preceded and followed by a silence or a change of speaker [24]. Sequence analyses study the meanings and the influence of a conversation element over the next one. For example, an utterance [25] or a behaviour [26].

Moreover, communication channels are evolving, and remote consultations are becoming increasingly common in today's healthcare. Digital technologies allow for synchronous (e.g. videocalls and chat messages) [27] and asynchronous consultations (e.g. non-instant messages, videos, voice messages) [28]. These modalities are especially useful for patients that live in remote areas [29] or for consultations that do not require a physical examination, such as psychological therapy [30] and lifestyle interventions [31]. Furthermore, evidence shows that telehealth alternatives can be as effective as face-to-face consultations [32,33]. The major questions are whether we can study them similarly and whether a linguistic analysis would add fresh perspectives in both contexts. Telehealth possibilities include video calls and messages with images, audio, or only text. These digital interactions can be either *synchronous* if the communication occurs in real time, such as calls and instant messaging, *asynchronous* if the messages are exchanged over a longer period, or a combination of the two.

Modern communication research has produced several verbal communication interaction coding systems [34]. However, only a few scholars apply them to written health interactions, primarily through conversation analysis (CA) [35,36]. Written communication has the major advantage of being faster to code than oral communication because there is no need for transcription and nonverbal cues are absent. Additionally, this format has unique features, such as message persistence in time and textual quotation in response to a specific message, which facilitate sequence analysis. As a result, language researchers developed digital CA [37]. But if we wish to go deeper into the communicative function, language production, and meanings, we can turn to Systemic functional linguistics (SFL) [38]. SFL is an analytical approach suitable for any kind of language production, including text analysis [39]. In SFL, three metafunctions construe the social meaning articulated by a speaker: ideational, which refers to the speaker's inner and outer experience, interpersonal, which concerns the interaction, such as the relationships between the speaker and their message as well as between the speaker and the recipient, and textual, which refers to the interpretation of the text as a text rather than just an array of words [39]. A transitivity analysis contributes to the ideational metafunction by investigating the syntax, participants, and circumstances of an utterance to comprehend the experience expressed by the speakers [40]. SFL is applicable to healthcare interactions [41], and only a few researchers have used it to investigate digital communication, such as emoji use [42].

Despite the increasing use of text-based interventions, there are no validated methods for analysing asynchronous health interactions to our knowledge. For these reasons, there is a need to expand interaction research to a teleconsultation setting and its characteristic features [43]. From both a qualitative and quantitative perspective, we have previously discussed the methodologies that are applicable to digital health interaction analysis [44]. In this article, we review the literature to identify existing coding systems for healthcare interactions. Furthermore, we investigate their application to written communication, along with the potential challenges that may arise in a digital setting.

## 2 Material and methods

### 2.1 Search process and study selection

We carried out a meta-narrative review by searching six databases: PubMed, PsycINFO, Embase, Web of Science, CINAHL, and Scopus. Our search terms used were Communicati* AND Human* AND Linguistic* AND Professional-Patient Relation*, based on the MeSH terms from two publications by Gainforth et al. [45,46]. We explored additional search terms such as ‘interaction’ and ‘healthcare’. However, these terms significantly reduced the search results without yielding any relevant articles. We applied a language restriction for English results with no date restrictions, including all papers published up until December 2022. We compared the retrieved results to remove duplicates. We screened the remaining publications for title and abstract, and lastly for full text according to the following inclusion criteria: 1) Healthcare interaction; 2) Assessment made by direct or indirect observation of the consultation (i.e., live or audio/videotape recording); 3) Dyadic interaction; 4) Measuring both physicians' and patients' utterances. ERV carried out the database searches and screening. ERV, TCS, and HSP independently assessed the relevant studies for full text and discussed their judgments until consensus for final inclusion. This review process complies with the RAMESES publication standards [47].

### 2.2 Tool classification

**Fig. 1:**
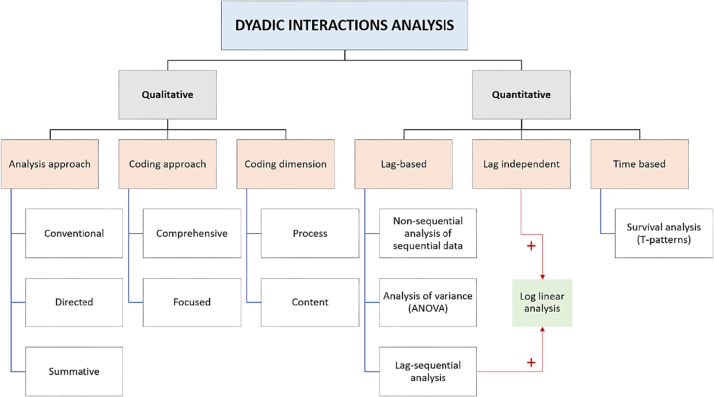
Classification of tools used in dyadic interaction analysis based on their methodology [44]. Qualitative methods, such as content analysis and conversation analysis (CA), are categorized according to their analytical approach, coding approach, coding dimension, and subcategories. Quantitative methods, including Roter's Interaction Analysis System (RIAS) and sequential analysis methodologies (lag-based, lag-independent, and time-based), are classified along with their respective subcategories.

We classified each tool according to their methodology as qualitative or quantitative. We used a classification framework for patient-provider interaction analysis methods from our previous work [44]. For those that were qualitative, we further classified them according to their analysis approach, coding approach, coding dimension, and their subcategories. For those tools that were quantitative, we classified them according to their sequential analysis methodology as lag-based, lag independent, and time-based, and their subcategories. We illustrate this classification in Fig. 1.

### 2.3 Data extraction

We reviewed all the articles several times and searched for studies using each specific tool. The data we extracted from the selected studies included a description of the coding tool, together with the unit of analysis (UoA), the behaviours measured, reliability, strengths, weaknesses, and further observations.

### 2.4 Reliability

We identified inter-rater reliability (IRR) scores for each tool. IRR is a widely used and accepted metric for assessing the trustworthiness of a qualitative coding approach [48]. It is calculated by dividing the number of agreed codes among coders by the total number of codes in the data set. A 0.70 reliability coefficient is considered an acceptable score for new code systems [49]. If this score was missing from the tool's description, we searched for the IRR reported elsewhere by the authors or for a study that applied the tool to a healthcare setting.

## 3 Results

### 3.1 Study selection

**Fig. 2:**
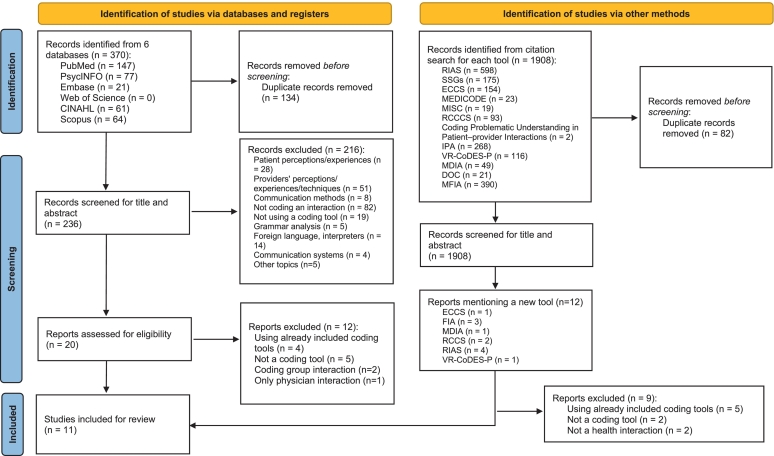
Flow diagram of the meta-narrative review search.

From the initial literature search, we identified 370 publications for review. After we searched for duplicates, 236 of these studies remained. Subsequently, we removed 216 citations during title and abstract screening. We reviewed the remaining 20 citations independently by full text, which resulted in the inclusion of 8 coding tools. Additionally, we conducted a citation search for each of these 8 tools, which resulted in the addition of 3 more tools to this review. We describe the data extraction process in Fig. 2, inspired by the PRISMA guidelines for flow diagrams when reporting systematic reviews [50]. Our search strategy identified additional coding tools that did not meet the specified inclusion criteria, and therefore, were not included.

### 3.2 Coding tools' characteristics

Table 1 shows the characteristics of the 11 coding tools that we identified published from 1973 to 2019. Most were developed in the US (*n* = 7), two in Europe (Italy and Portugal, respectively), one in Australia, and one cross-country [51]. The UoA was the utterance for most of the tools (n = 7), as well as patient-created empathic categories or empathic opportunities (EOs) and the physician's empathic responses (*n* = 2), speech segmentations for content and function (*n* = 1), and a triad consisting of an eliciting event, a cue or concern, and a response (n = 1).

**Table 1:** Summary of tool characteristics. EO: Empathic Opportunity, HPE: Health Provider-Elicited, PE: Patient-Elicited, HP: Health Provider.

Unit of analysis	Name of the tool	Author	Year	Country	Categories
Utterance	Relational Communication Control Coding System (RCCCS)	Ericson & Rogers	1973	US	First utterance according to grammatical format (assertion, question, or talkover) and responses according to pragmatic function (answer, order, or topic change) + control code (↑;domineering, ↓;submissive, and →;transitory/neutral)
	Roter's interaction analysis system (RIAS)	Roter	1977	US	29 task-focused categories for doctor (technically based skills: data gathering, tests, physical examination, patient education and counselling) and patient (need to know and understand: question–asking, information giving) + 14 socioemotional categories for doctor (social amenities, empathy, concern, reassurance) and patient (concern, optimism, empathy, joking, laughter, chit-chat) + 6-point scale for affective dimension (anger, anxiety, dominance, interest, friendliness)
	Verbal Response Modes (VRM) Coding System	Stiles	1992	US	Each utterance is coded twice according to its literal meaning and to its communicative intent or pragmatic meaning as reflection (R), acknowledgment (K), interpretation (I), question (Q), confirmation (C), edification (E), advisement (A), or disclosure (D)
	Cancer-specific interaction analysis system (CN-LOGIT)	Butow et al	1995	Australia	Number and sequence of events + source (doctor, patient, third party), process (open/closed questions, initiated statements, responses to questions), content (diagnosis, prognosis, treatment, medical history and presenting symptoms, other medical matters, social matters, other), and emotional tone (friendly/warm, tense/ anxious, sad/depressed, frustrated/angry, matter of fact)
	Motivational Interviewing Skill Code (MISC)	Miller	1997	US	Resistance to change (resistant or cooperative responses) and behaviour counts for therapist (advice, affirm, confront, direct, emphasize control, facilitate, filler, inform, question, raise concern, reflect, reframe, support, structure, warn) and client (ask, follow, resist change, change talk)
	The Verona Medical Interview Classification System (VR-MICS)	Del Piccolo et al	1999	Italy	VR-MICS/P: 21 process (cue, statement, expectation/opinion or question) and content (psychological, psychosocial, lifestyle, symptoms, impact on life functions, occasional behaviour, illness management) codes. VR-MICS/D: 22 process codes (information-gathering, patient-facilitating, patient-involving, patient-supporting, and patient education)
	The Coordination and Competence System (CACS)	McNeilis	2002	US	Message content (medical and non-medical topics), alignment (one speaker's ability to uptake on the other's message), and function (31 categories for the communicative tasks in the conversation). Same for patient and physician utterances.
	Coding Problematic Understanding in Patient–provider Interactions	Rossi&Macagno	2019	Portugal	Communicative issues (anticipation, declaration of lack of understanding, alternative semantic interpretation, alternative pragmatic interpretation, irrelevant or incoherent turn, and no uptake for both patient's and provider's utterances)
Emotional expressions and responses	Multidimensional Interaction Analysis (MDIA)	Charon & Greene	1994	US	EOs and empathic responses classification according to content (biomedical, personal habits, psychosocial, patient-physician relationship, and other) and interviewing behaviours (questioning, informing, and supportiveness) + global variables for both physician (i.e., engaged-indifferent, patient-abrupt) and patient (i.e., assertive-passive, relaxed-tense)
	Empathic Communication Coding System (ECCS)	Bylund & Makoul	2002	US	Patient EOs': emotional intensity (high to low). Physician empathic responses: level 0 (denial of patient perspective), level 1 (perfunctory recognition of patient perspective), level 2 (implicit recognition of patient perspective), level 3 (acknowledgement), level 4 (confirmation), level 5 (statement of shared feeling or experience)
	Verona coding definitions of emotional sequences (VR-CoDES)	Zimmermann & Del Piccolo	2011	Italy, Netherlands, Norway, Belgium, Germany, UK, Australia and US	HPE or PE cues/concerns and 17 categories for HP responses: non-explicit reduction (ignore, shutting down, information advise) or provision (silence, back channel, acknowledge, active invitation, implicit empathy) of space and explicit reduction (switching, post-poning, information advice, active blocking) or provision (content as acknowledgment or exploration and affective as acknowledgement, exploration, or empathy) of space

### 3.3 Methodology classification

We searched for publications that employed these coding tools to learn more about how they are used in practice (see Table 2). In all cases, the coding categories were qualitative, which were then analysed quantitatively. According to the qualitative sub classification provided in Fig. 1, the coding dimension was equally split in *focused* (*n* = 6) and *comprehensive* (*n* = 5). The *process-oriented* coding approach was present in all the tools (*n* = 11). Additionally, two of the tools were both *content* and *process* oriented (*n* = 2). Regarding the main quantitative methodologies used, these were descriptive statistics, such as category means, frequencies and percentages, ratios and scores, and cross-tabulations (*n* = 4), Chi square tests (n = 4), as well as *t*-test and Pearson correlation (n = 1), and ANOVA and the GSK approach (n = 1) to explain and predict the interaction patterns [52]. Only one publication [53] combined descriptive statistics, Chi-square test, and interactive analysis to identify lag patterns and statistical differences (General Sequential Querier, GSEQ).

**Table 2:** Summary of tool application. HPE: Health Provider-Elicited, PE: Patient-Elicited.

Unit of analysis	Tool	Coding approach	Coding dimension	Quantitative methodology	Inter-rater reliability (IRR) [89]
Utterance	Relational Communication Control Coding System (RCCCS)	Comprehensive	Process	ANOVA and GSK [52]	0.79
	Roter's interaction analysis system (RIAS)	Comprehensive	Process	Lag-independent. t-test, Pearson correlation [90]	0.65–0.85
	Verbal Response Modes (VRM) Coding System	Comprehensive	Process	Descriptive statistics [91]	0.95 for form and 0.85 for intent [55]
	Cancer-specific interaction analysis system (CN-LOGIT) (Cancode)	Focused	Process	Descriptive statistics	0.50 for function and 0.59 for content
	The Verona medical interview classification system (VR-MICS)	Focused	Process	Chi-square test	0.91 for division into speech units and 0.82 for coding
	The Coordination and Competence System (CACS)	Comprehensive	Content and process	Lag-based. Descriptive statistics + Interactive analyses to identify lag patterns and check for statistical differences (General Sequential Querier, GSEQ) + *Z*-scores and Chi-squares	0.80–0.83 for content, 1.00 for over-lapping talk, 0.68–0.72 for acknowledgment tokens, 0.76–0.85 for alignment, and 0.70–0.87 for function
	Motivational Interviewing Skill Code (MISC)	Focused	Process	Descriptive statistics	0.77–0.90 for therapist rating and 0.79–0.91 for client rating [68]
	Coding Problematic Understanding in Patient–provider Interactions	Focused	Process	Descriptive statistics + Chi-square tests	0.93–1.00
Emotional expressions and responses	Multidimensional Interaction Analysis (MDIA)	Focused	Content and process	Lag-independent. Chi-square test [92]	0.8–0.9
	Empathic Communication Coding System (ECCS)	Focused	Process	Descriptive statistics	0.8 for patient's empathic opportunity, 0.79 for patient's emotional intensity rating and 0.73 for physician's empathic communication behaviours
	Verona coding definitions of emotional sequences (VR-CoDES)	Focused	Process	Lag-based. Chi-square test and Cramer's V to indicate the degree of correlation	0.93 for providers' responses, 0.70 for patients' emotional cues and concerns, and 0.70 for the distinction between HPE and PE cue/concern

### 3.4 Unit of analysis and coding categories

We found similarities among the six tools in which the utterance was the UoA. The Relational Communication Control Coding System (RCCCS) [54] codes the utterances according to their *grammatical format* as either *assertion, question,* or *talkover*. Coders classify each utterance in relation to the previous one based on its *pragmatic function* as either *answer, order,* or *topic change*. Additionally, each utterance receives a control code being *↑;domineering* (e.g. talkover), *↓;submissive* (e.g. offering support), and →;transitory/neutral (extending the topic). This way, the coding stablishes a sequential pattern. The Verbal Response Modes (VRM) system [55] codes each utterance twice as well. First, according to its *literal meaning,* that depends on the *sentence form* (declarative, imperative, or interrogative) and the *subject* (1st, 2nd, or 3rd person). Second, according to its *communicative intent* or *pragmatic meaning* as *reflection (R), acknowledgment (K), interpretation (I), question (Q), confirmation (C), edification (E), advisement (A), or disclosure (D).* Each pragmatic meaning category receives a role dimension: Informativeness (C, E, A, D), Attentiveness (R, A, I, Q), Directiveness (I, Q, A, D), Acquiescence (R, A, C, E), Presumptuousness (R, I, C, A) and Unassumingness (A, E, Q, D). The proportion of coded utterances serves to calculate a dimension index.

The cancer-specific interaction analysis system (CN-LOGIT) entails three analysis stages: a micro-level analysis of the events in real time to preserve the sequence, an events count, and a macro-level analysis of the consultation. This system has four dimensions: *source* (doctor, patient, or third party), *process* (open and closed questions, initiated statements, and responses to questions), *content* (diagnosis, prognosis, treatment, medical history and presenting symptoms, other medical matters, social matters, and other), and *emotional tone* (friendly/warm, tense/ anxious, sad/depressed, frustrated/angry or matter of fact). Cancode [56] is an adaptation of CN-LOGIT with additional categories for a more comprehensive analysis.

Originally in Italian, the Verona Medical Interview Classification System (VR-MICS) defines two separate classifications for patient (VR-MICS/P) [57] and doctor (VR-MICS/D) [58] utterances with 21 and 22 categories, respectively. Del Piccolo et al. [51] published an English version. In the VR-MICS/P, coders classify patient's expressions according to the *process* as either *cue* or *statement*. Verbal (observation, question, comment) and non-verbal (tone of voice, expressed emotions such as crying) cues aim to draw the doctors' attention to a topic that has not been yet or sufficiently discussed. A statement is any utterance that connects with previous utterances and/or describes a cue. Other possible coding categories are *expectation/opinion* or *question asking*. Additionally, patient utterances are coded according to content (psychological, psychosocial, lifestyle, symptoms, impact on life functions, occasional behaviour, and illness management). The VR-MICS/D covers 22 mutually exclusive codes described in five process categories: *information-gathering interventions* (closed/open-ended questions, requests), *patient-facilitating interventions* (social conversation, facilitations, transitions), *patient-involving interventions* (asking for repetition, understanding, opinion or ideas), *patient supporting interventions* (agreement, reassurance, support, appraisal) and *patient education interventions* (information provision and instruction).

The Motivational Interviewing Skill Code (MISC) [59] is inspired by Chamberlain's [60] for assessing resistance to change in therapy. The MISC categories are *resistant responses* (1. Interrupt/talkover, 2. Negative attitude, 3. Challenge/confront, 4. Own agenda, 5. Not tracking) or *cooperative responses* (6. Nonresistant, 7. Facilitative). Additionally, this system codes the therapist's behaviour (*advice, affirm, confront…*) and the client's behaviour (*ask, follow, resist change, change talk*). The MISC also includes global evaluations of the therapist (*“acceptance”, “egalitarianism”, “empathy”, “genuineness”, “warmth”, and “spirit of MI”*), the client (*“affect”, “cooperation”, “disclosure”, and “engagement”*) and the interaction (*“collaboration”* and *“benefit”*) and measures the conversation and utterances' time length. In contrast, the Coordination and Competence System (CACS) [53] codes patient and physician's utterances separately according to the same categories of *message content* (medical and non-medical topics), *alignment* (speaker's ability to uptake on the other's message), and *function* (31 communicative tasks).

The Coding Problematic Understanding in Patient–provider Interactions system [61] is the only coding tool that focuses on the communicative challenges. There are six categories for both patient and provider's utterances: *anticipation, declaration of lack of understanding, alternative semantic interpretation, alternative pragmatic interpretation, irrelevant or incoherent turn,* and *no uptake*.

Lastly, the Roter's Interaction Analysis System (RIAS) [62] describes 29 task-focused categories for the doctor's (*technically based skills*, such as data gathering, physical examination, patient education and counselling) and the patient's (*need to know and understand*, such as question–asking and information-giving) utterances, and 14 socioemotional categories for the doctor (social amenities, empathy, concern…) and the patient (concern, optimism, joking…). All these categories are mutually exclusive and coded sequentially, including time annotation. In addition, each speaker receives a rating based on a 6-point scale for affective dimensions (anger, anxiety, dominance, interest, and friendliness).

Alternatively, three of the reviewed coding tools describe emotional expressions and their responses as the UoA and share a similar framework. The Empathic Communication Coding System (ECCS) [63] defines patient and physician's categories separately. Patient's EOs are a statement of emotion (‘*The surgery scares me*’), a statement of progress (‘*I have been eating healthy*’), or a statement of challenge (‘*I struggle to reach 10.000 steps*’). Each EO receives a 5-point scale rating according to its emotional intensity between 0 or low (e.g., statement or fact) and 5 or high (e.g., named emotion). Physician's responses to EOs are coded hierarchically: level 0 (denial of patient perspective), level 1 (perfunctory recognition of patient perspective), level 2 (implicit recognition of patient perspective), level 3 (acknowledgement), level 4 (confirmation), or level 5 (statement of shared feeling or experience). The ECCS considers that levels 3 to 5 correspond to an explicit recognition of the patient's perspective. Similarly, the Multidimensional Interaction Analysis (MDIA) [64] classifies patients' EOs and their empathic responses according to content (biomedical, personal habits, psychosocial, patient-physician relationship, other) and interviewing behaviours (questioning, informing, supportiveness). Additionally, MDIA includes global variables for both physician (i.e., engaged-indifferent, patient-abrupt) and patient (i.e., assertive-passive, relaxed-tense), and a classification for sentinel events or occurrences that merit special attention, such as *crying* or *prolonged silence*.

The Verona coding definitions of emotional sequences (VR-CoDES) system [65] considers a triad UoA: an eliciting event, a patient cue or concern, and a physician response. Since the authors of the VR-MICS contributed to the VR-CoDES, the coding categories share similarities [51]. A concern is an explicit expression of a negative emotion (*‘I am worried about the side effects’*), and a cue is an unclear expression of a negative emotion, such as words, phrases, or verbal hints to describe emotions (*‘I have been fired’, ‘This is a funny situation for me’*) [66]. Cues and concerns can be provider or patient-elicited, and health provider responses are classified into 17 subcategories according to the explicitness and the space provision for further disclosure, such as *asking for details*. Additionally, the VR-CoDES identifies conversation lags to establish a turns' sequence and distinguish between immediate and delayed HCP's responses to patients' cues and concerns [67].

Inter-rater reliability (IRR).

We show IRR scores in Table 2. With the exception of the MISC and VRM tools' IRR, defined elsewhere [55,68], the majority of original publications included this score. Frequently, IRR was represented as an overall score for all the categories that ranged from 0.65 to 0.85 [51,54,61,62,64]. Others assessed HCP and patient categories coding reliability separately that ranged from 0.73 to 0.93 and from 0.70 to 0.91, respectively [59,63,65]. In three of the tools, authors calculated IRR for the form, function, and/or content of the utterance [55,56]. Additional IRR scores evaluated the coding reliability of speech units division [51], distinction between categories (VR-CODES), and further categories such as acknowledgement tokens and alignment [53]. Overall, IRR scores were higher than 0.70. The lowest values were 0.50 and 0.59 for utterance function and content, respectively, in the Cancode interaction system, where authors suggested that the reason could be some overlapping coding categories [56].

## 4 Discussion and conclusion

### 4.1 Discussion

Our results show similarities across coding tools, whether these are utterances or emotional expressions and responses-based. As we anticipated, the coding categories concern conversation topics and behaviours. Some of the reviewed tools use different terminologies for the same concept, which are sometimes inconsistent with other frameworks. For example, the RCCCS and the VRM system code utterances twice: according to their *grammatical format* (RCCCS) or *literal meaning* (VRM) and according to the *pragmatic function* (RCCCS) or *communicative intent/pragmatic meaning* (VRM). Similarly, the syntax or structure encompasses the function or meaning of the message in SFL. According to the lexicogrammar approach, the lexis or vocabulary and the syntax combine into one and are dependent on each other [69]. These concepts correlate to the definitions of *syntactic* and *pragmatic* functions of language [70]. Moreover, there are two pragmatic functions within SFL's textual metafunction: *focus* and *topic*. The topic is the *theme* that the speakers discuss about, and the focus is the *rheme* or what they say about the topic [71]. As explained by Bolkestein [70], in question-answer patterns, the focus is in the answer. The RCCCS codes a question based on its syntactic function and an answer based on its pragmatic function. In the VRM system, a question and its response belong to the pragmatic function categories. Other frameworks, such as CA, code the question-answer structure as an adjacency pair. Sacks asserts that conversation turns are adjacent when they are associated, i.e., when a speaker shows understanding of the previous turn in the current turn [72].

The inconsistency in terminology also exists in the *process* and *content* categories. CN-LOGIT and VR-MICS use these exact terms, while MDIA only uses *content*. However, other coding systems use different terms for *process* such as behaviours (MISC, MDIA), communicative tasks (CACS), communication challenges (Coding Problematic Understanding in Patient-Provider Interactions system), HCP's responses (ECCS, VR-CoDES), and task-focused categories (RIAS). Similarly, the terms used to describe *content* categories are topics (CACS), type of statements in patient's EOs (emotion, progress, or challenge in ECCS), and socioemotional categories (empathy, concern, optimism, and laughter in RIAS).

There was a presence of emotional categories in 4 of the 11 coding tools, predominantly in the form of global evaluations of the HCP, the patient, and the interaction (MISC, MDIA), emotional intensity of patient's EOs (ECCS) and emotional tone (CN-LOGIT). The analysis of emotional phenomena can be beneficial because communication is a human phenomenon that extends beyond verbal exchanges. However, only 3 of the 11 tools (MDIA, ECCS, VR-CoDES) included emotional expressions and their responses as the UoA. While the content and actions of the conversation are relevant, research has proved the importance of HCP's emotional skills, such as empathy [73], and interpersonal skills, such as relationship building, to promote positive patient outcomes [74].

We identified a number of strengths and weaknesses for these coding tools. As we stated, all the coding tools include process-related categories. This is an overall strength. Although the topics discussed during a patient-provider consultation are relevant, Poole argues that the actions and the way these are performed shape the interaction and its outcome [76]. Rather than individual behaviour, the RCCCS codes the interaction in two-message UoA, which hinders a more extensive sequential analysis and the identification of interaction patterns. To address this, the authors of the RCCCS proposed an intensity measure, i.e., types of responses to a question, and a more precise time dimension, i.e., HCP's response time to a question [54]. Additionally, this tool is intended for group interaction analysis, particularly family relationships. Although some authors have used RCCCS for patient-provider interaction analysis, they determined that more dimensions are needed to understand the full picture of a dyadic interaction [77]. In contrast, the VRM [55] system is valid for both individual and group interactions: healthcare consultations, political speeches, or teaching lectures, and for written and spoken interactions. However, VRM categories are neither exhaustive nor mutually exclusive, and as stated by its authors, coders resolve overlapping categories by arbitrary rules of priority [55], with a strong influence on the results of the coding categories. One way to address this issue would be to provide clear priority guidelines to contribute to this tool's validity, IRR, and reproducibility. Still, although VRM's IRR scores were high (0.95 for form and 0.85 for intent) and they have reliably applied it to health interactions [78], its validity evidence is limited since a scarce number of studies have implemented it. Additionally, the discourses' variability in terms of audience, topic, objective, language, and channel may call for specialised coding. SFL can account for this via the concepts of register and genre, as well as claim that linguistic realisations are a product of their context. Some tools are specific to the constructs addressed, such as group process systems, arguments, personality and team behaviour, or roles and relationships [79]. In our review, only CN-LOGIT was tailored to cancer patients [56], with very low IRR scores (0.50 for function and 0.59 for content). The authors cited time constraints associated with training (several weeks) and coding (5 times the length of the consultation) as possible explanations. This is not an issue with RIAS, since coders work directly with the recording in the RIAS software in a hybrid scheme that covers a range of interactional variables, content and process-based.

Furthermore, RIAS categories are intuitive and can be adapted to different contexts (multiple speakers, task, and content-based communication) and languages (Polish, Rumanian, Estonian, Swedish, Japanese, Dutch). Evidence supports RIAS reliability and validity, and it can predict patient outcomes [62]. However, there are some weaknesses: the lack of transcription overlooks a large amount of analytical and interpretational work that lies in the transcription process; coding categories may overlap and do not account for interruptions or crying, whereas they do for laughing, and the absence of sequential analysis. Roter acknowledges that although lags are recorded during the coding process, the interaction analysis does not incorporate them further [62]: RIAS does not evaluate turn-taking or responses to specific questions. Although MDIA integrates multiple levels of the interaction (i.e., technical and narrative aspects of the health interaction), CN-LOGIT counts the number of events, and MISC measures the length of the conversation and each utterance, these variables are not valid for sequential analyses either. CACS and VR-CoDES, on the other hand, are the only coding tools that use lag-records and account for sequentiality in the conversation. Unfortunately, CACS showed low reliability, which McNeilis attributed to insufficient coder training and/or motivation, and a lack of category specificity [53]. The problem with sequentiality in coding tools that use emotional categories, such as ECCS and MISC, is that they count and assess observable behaviours as a whole, rather than as processes that follow each other. A number of utterances may be necessary to fully grasp some concepts, such as empathy, as well as how they relate to one another. Although the VR-CoDES coder indicates who initiates the prior statement (health provider or patient), this tool shares the same issue in that this system focuses on HCP responses rather than on the content of prior statements to cues and concerns.

Nearly all existing coding tools create categories based on interpretation of the conversation, behaviours, or themes rather than grammar and socioemotional categories. Our results suggest that a linguistic analysis, in addition to sequentiality [34], may be what is lacking in interaction analysis research. The study of grammar digs deeper into the conversation's events and the interpretation and exchange of meanings [80]. Although it has been widely applied to verbal health interactions, less so in a written context [81]. The reviewed tools can be partially applied to written interactions due to the common use of transcriptions before coding, and could benefit from a linguistic analysis. Some important paralinguistic features (e.g., tone, gestures, facial expressions) provide valuable information about the interaction, and are absent or substituted in written communication (e.g., emojis and capital letters). However, as highlighted in the Introduction section, researchers often resort to other methods such as digital CA [35] and data mining [75]. Therefore, there is a significant need for studies that apply oral communication coding tools and linguistics to this setting and explore how to adapt them accordingly.

Based on SFL, Pounds [82] developed the empathy ‘appraisal’ approach for asynchronous HCP-patient interactions. An appraisal is an evaluative interpretation of a situation [83], and our empathic emotions are the result of our interpretation of the other person's situation, not our perception of it [84]. Appraisal theories make three general claims about our emotions as interpretations of our own situations: first, our appraisals determine our emotions, such as the *anticipated effort* or the amount of effort required to complete a task. Second, the boundaries between emotions are continuous; e.g., that anticipated effort may make us angry, but anger can take many forms and imply other emotions such as fear and sadness. Third, emotions have universal appraisal patterns, which means that if two people evaluate situations in the same way, regardless of whether they are similar or not, they will express the same emotion [85]. Wondra and Ellsworth developed the *appraisal theory of empathy* as a bridge between emotion and empathy theories based on these claims, particularly the last one. They argue that if two people appraise a situation similarly, they will experience the same emotion, which will elicit empathy [86]. Pounds' framework is the first to apply the concept of empathy appraisal to healthcare interactions using a linguistic approach. Furthermore, she and De Pablos-Ortega [87] applied it to asynchronous health interactions, which is the issue we address in this article, with promising results. This is a significant analytical step, and by informing it further with SFL, we can at least partially account for the intermediate steps between expression/utterance and interpretation. Although we contacted Pounds and De Pablos-Ortega, they confirmed that they are no longer involved in this empathy appraisal work. Therefore, we conducted a pilot study that combined empathy appraisal and SFL, with successful findings, which suggest that these two approaches complement each other [88]. This novel approach could supplement the coding tools that we review in this article and provide a complete picture of the interaction.

Our study has several strengths. Firstly, we conducted a comprehensive search process that covered multiple databases and incorporated relevant MeSH terms, ensuring a thorough approach to capturing relevant studies. Secondly, we adhered to the RAMESES publication standards for meta-narrative reviews, ensuring a systematic and transparent methodology. Lastly, we used a classification framework for dyadic interaction analysis methods that facilitated a structured and consistent categorization of the identified coding tools. However, our study also has limitations. Firstly, the reliance on a single researcher for screening titles and abstracts may have affected the robustness of our search strategy. Secondly, our search was limited to English-language publications, potentially introducing language bias and excluding relevant coding tools in other languages. Additionally, the heterogeneity of coding tools in terms of methodology, UoA, and coding categories presented challenges in synthesizing findings and drawing definitive conclusions. Finally, IRR data was missing or not explicitly provided for some of the coding tools, which limited the assessment of their reliability. Despite these limitations, our study offers valuable insights into the topic and serves as a foundation for future research in the field.

### 4.2 Innovation

This article represents the most comprehensive and up-to-date overview of coding tools for dyadic health interactions. Our findings highlight the one-sided nature of coding categorization in healthcare interactions, focusing primarily on value-centered evidence-based applications. This approach overlooks various aspects of the overall conversation with the patient. We anticipate that a holistic, empathy-based interdisciplinary approach will enhance patient-centered care and the patient-HCP relationship. Our research aims to support communication researchers in selecting appropriate coding tools amidst the diverse methodologies and terminologies available. With the rise of telehealth consultations, it is crucial to adapt interaction analysis methods for written consultations. Our empathy appraisal and linguistics model may improve interpretation, evaluation, and fidelity assessment of patient-HCP interactions, identifying areas for improvement and optimizing patient outcomes. These insights are valuable for future HCP training.

### 4.3 Conclusion

Our review shows that patient-provider communication coding tools overlook the development of healthcare interactions and the patients' responses. Despite similarities, the coding categories terminologies were inconsistent. Their focus mostly covered conversation topics and interaction behaviours. While these elements are relevant in health interactions' analysis, we propose a coding tool with emotional and grammar categories that could bring novel insights into patient-HCP communication. Furthermore, there is a pressing need to apply oral communication coding tools to written health interactions in future studies and explore their adaptations and limitations.

## Author contributions

ERV conducted the meta-narrative review, synthesized the results, and prepared and edited the manuscript until submission. HSP contributed to the data analysis and interpretation processes and participated in the manuscript reviewing process. DHL reviewed the final manuscript. TS conceived the research, supported the interpretation and discussion of results, and reviewed the final manuscript. All authors contributed to the article and approved the submitted version.

## Registration and protocol

We did not prepare a protocol review, and we did not register this review.

## Funding

This research is part of an Industrial PhD project sponsored by 10.13039/100012774Innovation Fund Denmark, the 10.13039/501100001734University of Copenhagen, and Liva Healthcare.

## Declaration of Competing Interest

The authors declare that the research was conducted in the absence of any commercial or financial relationships that could be construed as a potential conflict of interest.
